# Identification of potential biomarkers for diabetic nephropathy via UPLC-MS/MS-based metabolomics

**DOI:** 10.3389/fendo.2025.1581691

**Published:** 2025-09-01

**Authors:** Hetao Chen, Peipei Du, Tao Jiang, Ying Li, Yuanyuan Li, Yalin Liu, Baotong Yang, Jingyi Kang, Jiajia Duan, Yujin Ma, Xiangmei Chen, Hongwei Jiang

**Affiliations:** ^1^ Department of Clinical Laboratory, The First Affiliated Hospital, and College of Clinical Medicine of Henan University of Science and Technology, Luoyang, China; ^2^ Department of Pharmacy, The First Affiliated Hospital, and College of Clinical Medicine of Henan University of Science and Technology, Luoyang, China; ^3^ Department of Central Laboratory, The First Affiliated Hospital, and College of Clinical Medicine of Henan University of Science and Technology, Luoyang, China; ^4^ Henan Key Laboratory of Rare Diseases, Endocrinology and Metabolism Center, The First Affiliated Hospital, and College of Clinical Medicine of Henan University of Science and Technology, Luoyang, China; ^5^ Senior Department of Nephrology, the First Medical Center of Chinese People’s Liberation Army (PLA) General Hospital, Chinese People’s Liberation Army (PLA) Institute of Nephrology, National Key Laboratory of Kidney Diseases, National Clinical Research Center for Kidney Diseases, Beijing Key Laboratory of Kidney Diseases Research, Beijing, China

**Keywords:** diabetic nephropathy, metabolites, serum, biomarkers, UPLC-MS/MS

## Abstract

**Objective:**

Diabetes mellitus (DM) is a prevalent chronic disease, with diabetic nephropathy (DN) being a significant complication. Early detection of DN is critical for effective management. Current diagnostic methods, such as urinary albumin-to-creatinine ratio (uACR) and estimated glomerular filtration rate (eGFR), have limitations. Metabolomics offers a promising alternative by identifying specific metabolic signatures associated with DM and DN. This study aimed to identify potential metabolic biomarkers of DN using metabolomics.

**Methods:**

A total of 100 participants were recruited, including 20 healthy controls and 80 DM patients, who were classified into three groups based on uACR: normoalbuminuria (DM), microalbuminuria (DN-1), and macroalbuminuria (DN-2). Metabolomic profiles were analyzed using ultra-high performance liquid chromatography-tandem mass spectrometry (UPLC-MS/MS).

**Results:**

Results showed 74, 86, and 107 differentially expressed metabolites in the DM, DN-1, and DN-2 groups, respectively, compared to healthy controls. Compared to the DM group, DN-1 had 70 differential metabolites (55 upregulated, 15 downregulated), and DN-2 had 91 (81 upregulated, 10 downregulated). Between DN-1 and DN-2, 71 differential metabolites were identified (57 upregulated, 14 downregulated). Key metabolites such as lactate, L-ornithine, L-tryptophan, L-alanine, adenine, and cholecalciferol emerged as potential biomarkers and therapeutic targets. Venn diagram analysis identified 36 common differential metabolites across all groups. KEGG enrichment analysis highlighted significant involvement of amino acid biosynthesis and arginine and proline metabolism pathways in DN.

**Conclusion:**

In conclusion, this study provides valuable insights into potential metabolic markers and mechanisms for early identification and prediction of DN progression, which may aid in developing more accurate diagnostic tools and targeted therapies for DN.

## 1 Introduction

Diabetes mellitus (DM) is a common chronic disease that affects millions of people worldwide, resulting in poor health outcomes and escalating healthcare costs (1). Diabetic nephropathy (DN), one of the most severe complications of DM, is the leading cause of end-stage kidney disease (ESKD) and is now recognized as the fourth leading cause of death globally (2). Studies have shown that DN develops in 30-40% of individuals with diabetes, and over 50% of ESKD cases are attributed to DN (3–7). Therefore, timely identification of DN is crucial for effective prevention and management.

However, current clinical diagnosis and staging of DN primarily rely on the urine albumin-to-creatinine ratio (ACR) and estimated glomerular filtration rate (eGFR). Albuminuria is considered a highly specific marker for diagnosing kidney disease because it can indicate kidney damage even before a decline in glomerular filtration (8). However, clinically, renal injury in DN patients may have been ongoing for a considerable duration prior to significant changes in urinary albumin levels. Furthermore, normoalbuminuric diabetic kidney disease (NADKD) is prevalent. Additionally, reversing the condition after the onset of proteinuria is relatively challenging. Therefore, the sensitivity and specificity of urinary albumin, particularly microalbuminuria, are limited, posing challenges for early diagnosis and appropriate classification of chronic kidney disease (CKD) in clinical settings.

In the past decade, high-throughput metabolomic techniques have provided critical insights into the preconditions and pathophysiological pathways of diabetes, facilitating their widespread application in the clinical diagnosis of DN (4, 9–11). Liquid chromatography-mass spectrometry (LC-MS)-based metabolomics has become a prevalent tool for monitoring metabolic changes in diabetes and predicting disease progression. Numerous metabolomic studies have demonstrated that serum metabolites are significantly altered in patients with DN and type 2 diabetes mellitus (T2DM), including sugar metabolites and derivatives, amino acids (such as aromatic amino acids, glycine, glutamine, and glutamate), alpha-hydroxybutyrate (α-HB), hexadecanoic acid (C16:0), linolelaidic acid (C18:2n6t), and linoleic acid (C18:2n6c) (10, 12–14). These alterations in serum metabolites enhance our understanding of the metabolic mechanisms underlying T2DM onset and progression and aid in identifying early potential metabolic markers (15–17).

The identification of definitive markers for accurately estimating the stages of DN in patients is crucial. Early detection and timely intervention can significantly mitigate the risk of kidney injury in DN patients. Consequently, enhancing the detection capability for DN and identifying more sensitive and specific biomarkers are essential to facilitate early diagnosis and predict disease progression. In this study, we utilized ultra-performance liquid chromatography-tandem mass spectrometry (UPLC-MS/MS) to analyze the metabolomic profiles of healthy individuals and those with T2DM, aiming to identify novel metabolic markers indicative of DN in diabetic patients. Additionally, we investigated the correlation between differential metabolites as potential predictors of DN progression.

## 2 Materials and methods

### 2.1 Ethics statement

The written informed consent was obtained from all subjects, and the study design was approved by the Ethics Committee of the First Affiliated Hospital of Henan University of Science and Technology. The ethical approval number for this study is 2024-0585. The registration number at the Chinese Clinical Trial Registry (ChiCTR) is ChiCTR2400093494.

### 2.2 Sample collection

A total of 100 participants (61 males and 39 females) were included in this study, consisting of 20 healthy volunteers and 80 patients with T2DM. The average age was 53.62 ± 13.24 years. All samples were from the first affiliated hospital of Henan university of science and technology. Inclusion criteria included (1): age ≥ 18 years (2); clinical diagnosis of DM, which was in line with the American Diabetes Association (ADA) criteria (18). Exclusion criteria included (1): age <18 years (2); T1DM; (3) suffering from diseases that affected urinary albumin secretion and eGFR, such as benign and malignant tumors, hypertension, and urogenital infections; (4) kidney transplantation; (5) menstruating, pregnant, and lactating women. According to the KDIGO guidelines, patients with type 2 diabetes were divided into three groups: DM (uACR <30 mg/g), DN-1 (uACR 30–300 mg/g), and DN-2 (uACR >300 mg/g) (19). Healthy volunteers were used as control in this study. Blood samples were collected the next morning after fasting. Blood samples were centrifuged to prepare serum samples, which were then frozen at -80°C.

### 2.3 Metabolomic analysis

#### 2.3.1 Serum sample pretreatment

All serum samples were treated as previously described (20). The samples were thawed in ice water bath and was mixed by vortexing for 30s. Two hundred fifty microliters of water and 1200 µL acetonitrile-methanol (1:1, containing isotope internal standards) (CNW Technologies) were added into 50 µL sample and were vortexed for 30 s. After sonication in ice-water bath for 15min, samples were incubated at -40°C for 2 h. Then the samples were centrifuged at 12000 rpm and 4°C for 15 min, 1200 µL supernatant of each sample was transferred to a new Eppendorf tube and was dried with a centrifugal concentrator. One hundred twenty microliters acetonitrile (60%) was added into the Eppendorf tube to reconstitute the dried samples. The Eppendorf tube was vortexed until the powder was completely dissolved, followed by centrifugation at 12,000 rpm for 15 min at 4°C.

#### 2.3.2 UPLC–MS/MS

The supernatant of each sample (60-70 µL) was transferred to glass vial for LC-MS/MS analysis. Mixture of standard metabolites were prepared as QC sample. The LC separation was carried out using an UPLC System (1290, Agilent), equipped with a Waters Atlantis Premier BEH Z-HILIC Column. Mobile phase A consisted of a mixture of H_2_O and acetonitrile (9:1), containing 10 mmol/L ammonium formate, while mobile phase B consisted of a mixture of H_2_O and acetonitrile (1:9), also containing 10 mmol/L ammonium formate. The auto-sampler temperature was set at 4°C and the injection volume was 1 µL. AB Sciex QTrap 6500 plus mass spectrometer was applied for assay development. Typical ion source parameters were as follows: IonSpray Voltage: +5500V/-4500V, Curtain Gas: 35 psi, Temperature: 400°C, Ion Source Gas 1: 50 psi, Ion Source Gas 2: 50psi.

Raw data files generated by LC-MS/MS were processed with SCIEX Analyst Work Station Software (1.7.3). Metabolites quantification was analyzed with BIOTREEBioBud (2.0.3). Multivariate analysis, including fold change, principal component analysis (PCA) and orthogonal partial least-squares discriminant analysis (OPLS-DA) and variable importance in the projection (VIP) values, were performed on the SIMCAV18.0.1 software package (Sartorius Stedim Data Analytics AB, Umea, Sweden). Heatmap of hierarchical clustering analysis was conducted in R (ggplot2) 3.3.5 package (Vienna, Austria).

### 2.4 Statistical analysis

SPSS25.0 was used for statistical analysis. The continuous variables of clinical characteristics among the study population were presented as means ± standard deviations for normally distributed data, while categorical variables were reported as frequencies. The chi-squared test was used for categorical variables. To ensure the validity of the student’s t-test, we rigorously verified the underlying assumptions, including the normality of distribution and homogeneity of variance between groups. For data conforming to both normal distribution and homogeneity of variance, Student’s t-tests were conducted. In cases where data exhibited normal distribution but heterogeneity of variance, Welch’s t-tests were applied. For data that did not meet the assumption of normal distribution, non-parametric tests (Mann-Whitney tests) were utilized. One-way ANOVA tests were conducted for data that adhered to normal distribution and homogeneity of variance. For data that exhibited normal distribution but heterogeneity of variance, Welch’s ANOVA tests were performed, with multiple comparisons corrected using the Dunnett T3 method. Non-parametric tests (Kruskal-Wallis tests) were applied to data that did not conform to normal distribution, with Dunn’s test used for *post-hoc* corrections. *P <*0.05 was considered statistically significant.

The metabolites with VIP >1 and *P <*0.05 were considered as significantly changed metabolites. Area Under Curve with values 0.8 displayed a very high prediction effect of identified metabolites on disease.

## 3 Result

### 3.1 Clinical data of population

Clinical data characteristics of 100 subjects were shown in **Table 1**. Twenty individuals were healthy, with an average age of 43.95 ± 8.47 years. Of these, ten were male and ten were female. As for patients with T2DM, the average age was 48.6, 57.2, 61.1 years among three groups, respectively. Data showed that there was a significant difference in age among control group, DM group, DN-1 group and DN-2 group (*P* < 0.05), and DN-2 group was significantly older than the others. Furthermore, there was no significant difference in sex among the four groups (*P <* 0.05) (**Table 1**). There were significant differences in drinking and smoking habits among the three groups (*P* < 0.05), suggesting that these factors may serve as independent contributors to DN. There was a significant difference in UACR among three groups (*P* < 0.05), the mean levels were 2.22 mg/g, 118.04 mg/g and 2183.37 mg/g, respectively. Furthermore, statistically significant differences were observed in u-AlB, CREA, LDH, and eGFR among the three disease groups (**Table 1**). The mean levels of u-ALB, CREA and LDH were the highest in the DN-2 group, the mean eGFR of the DN-2 patients was 49.96 mL/min/1.73m^2^. This suggested that the kidney damage is severely and renal function was significantly impaired. In addition, the duration of diabetes and age at diabetes onset in patients with DN was significantly difference than that in patients with diabetes (*P* < 0.05); the average of the latter was 7.78 years and 40.96 years. In terms of blood glucose, HbA1c, ALT, URCA, CHO, LDL, HDL and BMI, there was no significant difference between patients with diabetes and patients with DN (*P* > 0.05, **Table 1**). However, the contents of blood glucose in three diabetes group were significantly higher than that in healthy group (*P* < 0.05) (**Table 1**). Compared to control group, the levels of TG, AST were significantly higher in DN-2 group (*P* < 0.05).

**Table 1:** Clinical characteristics of enrolled patients.

clinical features	Control(n=20)	DM(n=30)	DN-1(n=30)	DN-2(n=20)	*P* value
Age (years)	43.95 ± 8.47	48.6 ± 10.7	57.2 ± 14.95^Δ^	61.1± 10.73^Δ^	*P*<0.05
Sex (male/female)	10/10	20/10	15/15	10/10	0.502
Drinking (yes/no)	–	14/16	21/9	2/18	*P*<0.05
Smoking (yes/no)	–	15/15	11/19	3/17	*P*<0.05
u-AlB (mg/L)	–	7.09 ± 4.21	329.77 ± 427.89^Δ^	1793.98 ± 1140.03^Δ◇^	*P*<0.05
uACR (mg/g)	–	2.22 ± 3.46	118.04 ± 76.82^Δ^	2183.37 ± 2284.33^Δ◇^	*P*<0.05
CREA (umol/L)	–	55.27 ± 10.02	71.73 ± 33.01^Δ^	171.8 ± 122.18^Δ◇^	*P*<0.05
LDH (U/L)	–	131.27 ± 12.82	180.87 ± 39.95^Δ^	225.45 ± 56.23^Δ◇^	*P*<0.05
eGFR (mL/min/1.73m^2^)	–	115.15 ± 10.46	95.44 ± 29.14^Δ^	49.96 ± 43.85^Δ◇^	*P*<0.05
Blood glucose (mmol/L)	5.04 ± 0.34	7.36 ± 3.07^◆^	7.99 ± 5.97^◆^	8.99 ± 2.05^◆^	*P*<0.05
HbA1c (%)	–	8.75 ± 2.15	9.08 ± 3.06	8.99 ± 2.05	0.527
BMI (kg/m^2^)	23.8 ± 2.48	23.19 ± 2.71	24.07 ± 5.46	24.07 ± 6.56	0.748
Age at diabetes onset (years)	–	40.96 ± 10.59	44.23 ± 11.88	49.7 ± 10.48^Δ^	*P*<0.05
Diabetes duration (years)	–	7.78 ± 6.87	12.99 ± 7.65^Δ^	11.41 ± 6.46	*P*<0.05
ALT (U/L)	18.95 ± 7.35	19.8 ± 11.32	20 ± 14.76	19.73 ± 14.84	0.648
AST (U/L)	19.57 ± 5.62	16.83 ± 5.03	20.33 ± 10.9	20.57 ± 7.61^◆^	*P*<0.05
UREA (mmol/L)	4.29 ± 1.08	5.8 ± 1.59	7.17 ± 3.84	11.54 ± 6.99^◆^	*P*<0.05
URCA (µmol/L)	319.35 ± 70.40	301.6 ± 98.63	348.33 ± 100.24	394.84 ± 147.05	0.053
CHO (mmol/L)	4.99 ± 0.85	4.33 ± 1.0	4.65 ± 2.04	5.4 ± 2.07	0.414
TG mmol/L)	1.17 ± 0.37	1.99 ± 1.6	2.42 ± 2.29	2.23 ± 1.22^◆^	*P*<0.05
LDLC (mmol/L)	2.67 ± 0.6	2.44 ± 0.75	2.40 ± 0.78	2.82 ± 1.16	0.631
HDLC (mmol/L)	1.28 ± 0.21	1.07 ± 0.3	1.04 ± 0.39	1.11 ± 0.38	0.092

BMI, body mass index; eGFR, estimated glomerular filtration rate; uALB, urinary microalbumin; CREA, creatinine; HbA1c, glycated hemoglobin; CHO, cholesterol; TG, total triglycerides; HDLC, high-density lipoprotein; LDLC, low-density lipoprotein; uACR, urine albumin/creatinine ratio; ALT, alanine aminotransferase; AST, aspartate aminotransferase; LDH, lactate dehydrogenase; URCA, uric acid; ^Δ^ Compared with DM group, *P* < 0.05; ^◇^ Compared with DN-1 group, *P* < 0.05; ^◆^Compared with control group, *P* < 0.05.

### 3.2 Alterations in serum metabolic profiles between disease and health groups

**Figure 1:**
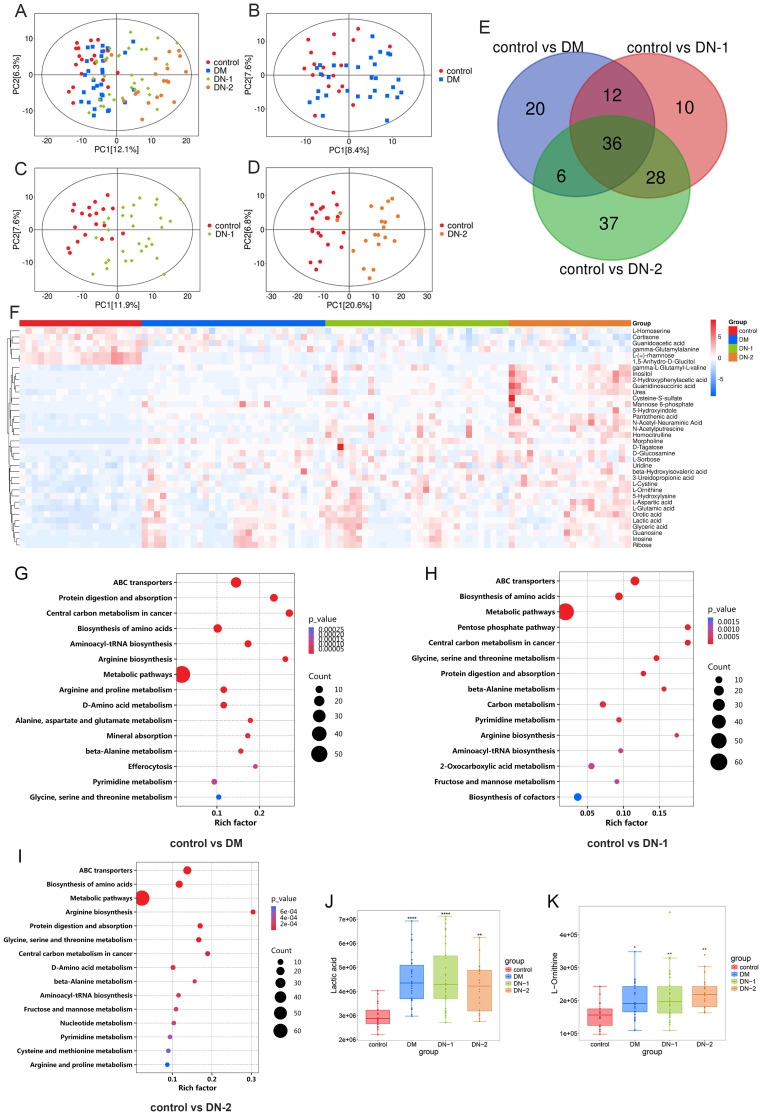
Metabolites composition and differences between disease and control groups. **(A)** PCA analysis of control, DM, DN-1 and DN-2 group; **(B)** PCA analysis of control vs. DM; **(C)** PCA analysis of control vs. DN-1; **(D)** PCA analysis of control vs. DN-2; **(E)** Venn diagram screening for common differential metabolites; **(F)** Heat map of the 36 differential metabolites; G-I. KEGG enrichment analysis of control vs. DM, control vs. DN-1 and control vs. DN-2; J-K. Expression levels of differential metabolites in different groups. *P < 0.05, **P < 0.01, ***P < 0.001, ****P < 0.0001.

Metabolomics analysis is a systems biology approach that provides comprehensive metabolic information from biological samples. This method has been widely applied in the diagnosis and treatment of diabetes and its complications. To assess the metabolic profile changes between DM, DN, and normal health individuals, we conducted quantitative targeted metabolomics analysis using serum samples from each group. In the PCA plot, no significant separation was observed between the DM and control groups, but there were differences in the distribution trends of the two groups (**Figure 1B**). Additionally, we observed a clear separation between the DN-1 and DN-2 groups and the control group, with the separation of the DN-2 group being more pronounced (**Figures 1C, D**). This indicates that there are significant changes in the metabolic profiles between each disease group and the normal group, particularly in the DN groups (**Figures 1A-D**). Compared to the control group, there were 74 different metabolites in the DM group, among which 51 were obviously up-regulated and 23 were obviously down-regulated. Compared to the control group, there were 86 different metabolites in the DN-1 group, among which 71 were obviously up-regulated and 15 were obviously down-regulated. Compared to the control group, there were 107 different metabolites in the DN-2 group, among which 95 were obviously up-regulated and 12 were obviously down-regulated. Utilizing the Draw Venn Diagram platform for the intersection screening of differential metabolites among four groups, we identified 36 common differential metabolites in the three disease groups compared to the normal group (**Figure 1E**, **Supplementary Table S1**). The heatmap illustrates the expression profiles of these differentially expressed metabolites across all groups (**Figure 1F**). Among the metabolites that exhibited a high correlation with DN were lactic acid and L-ornithine, and their expression levels across different groups are depicted in **Figures 1J, K**, respectively. KEGG enrichment analysis revealed the top 15 differentially enriched metabolic pathways between each disease group and control group. Notably, the shared differential metabolic pathways among all disease groups compared to the control group included “Biosynthesis of amino acids,” “Arginine and proline metabolism,” and “Glycine, serine, and threonine metabolism” (**Figures 1G, H**).

### 3.3 Alterations in serum metabolic profiles between DN and DM groups

**Figure 2:**
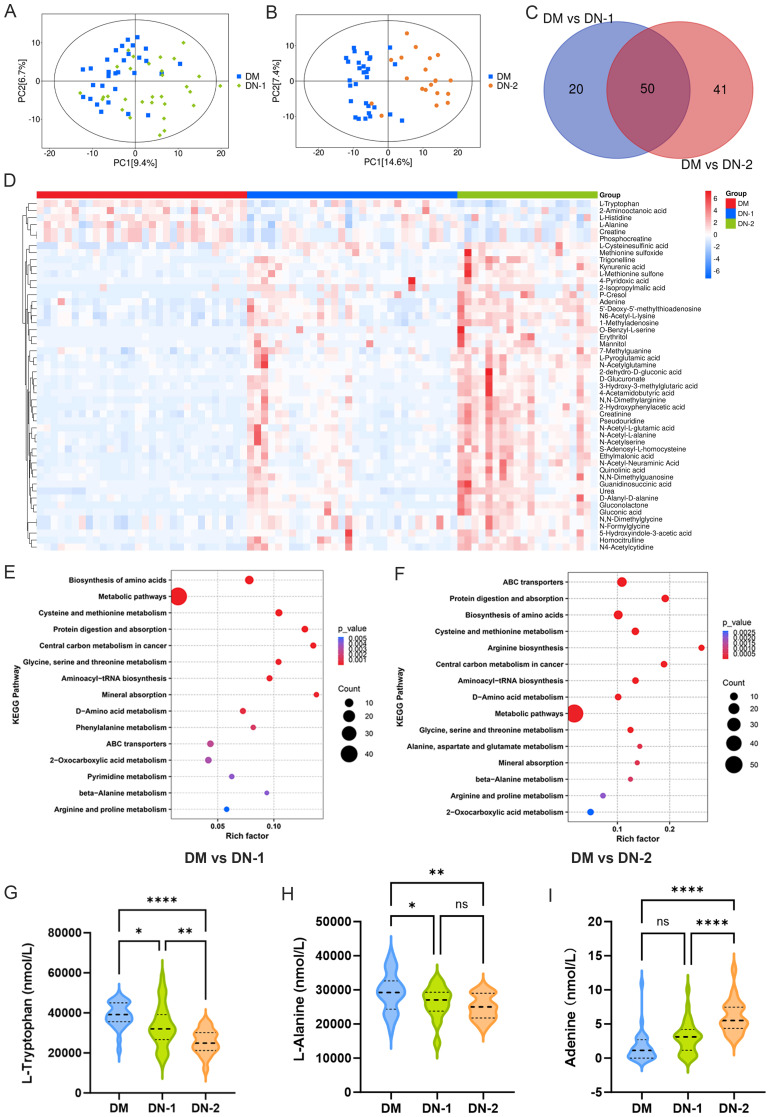
Metabolites composition and differences between diabetic nephropathy group and diabetic group. **(A)** PCA analysis of DM vs. DN-1; **(B)** PCA analysis of DM vs. DN-2; **(C)** Venn diagram screening for common differential metabolites; **(D)** Heat map of the 50 differential metabolites; E-F. KEGG enrichment analysis of DM vs. DN-1 and DM vs. DN-2; G-I. Expression levels of differential metabolites in different groups. *P < 0.05, **P < 0.01, ***P < 0.001, ****P < 0.0001.

To understand the differences in metabolites between DN and DM, we statistically analyzed each DN group against the DM group. PCA showed notable changes in metabolites for both DN-1 and DN-2 compared to DM (**Figures 2A, B**). The metabolic profiling results revealed that, compared to the DM group, the DN-1 group exhibited 70 differential metabolites, comprising 55 upregulated and 15 downregulated metabolites. Moreover, the DN-2 group displayed 91 differential metabolites, with 81 upregulated and 10 downregulated metabolites. We employed a Venn diagram analysis to identify 50 common differentially expressed metabolites in DN-1 and DN-2 groups relative to the DM group (**Figure 2C**). The detailed information regarding these metabolites is provided in **Supplementary Table S2**. The volcano plot illustrates the expression profiles of the 50 differentially expressed metabolites across the three groups (**Figure 2D**). KEGG enrichment analysis revealed the top 15 significantly altered pathways in the comparisons of DM vs. DN-1 and DM vs. DN-2, respectively (**Figures 2E, F**). Notably, the commonly affected pathways included “Biosynthesis of amino acids”, “Cysteine and methionine metabolism” and “beta-Alanine metabolism” Furthermore, three metabolites associated with DN—L-tryptophan, L-alanine and adenine—were identified, and their expression levels in each group were visualized using violin plots (**Figures 2G-I**).

### 3.4 Alterations in serum metabolic profiles between DN-1 and DN-2 groups

**Figure 3:**
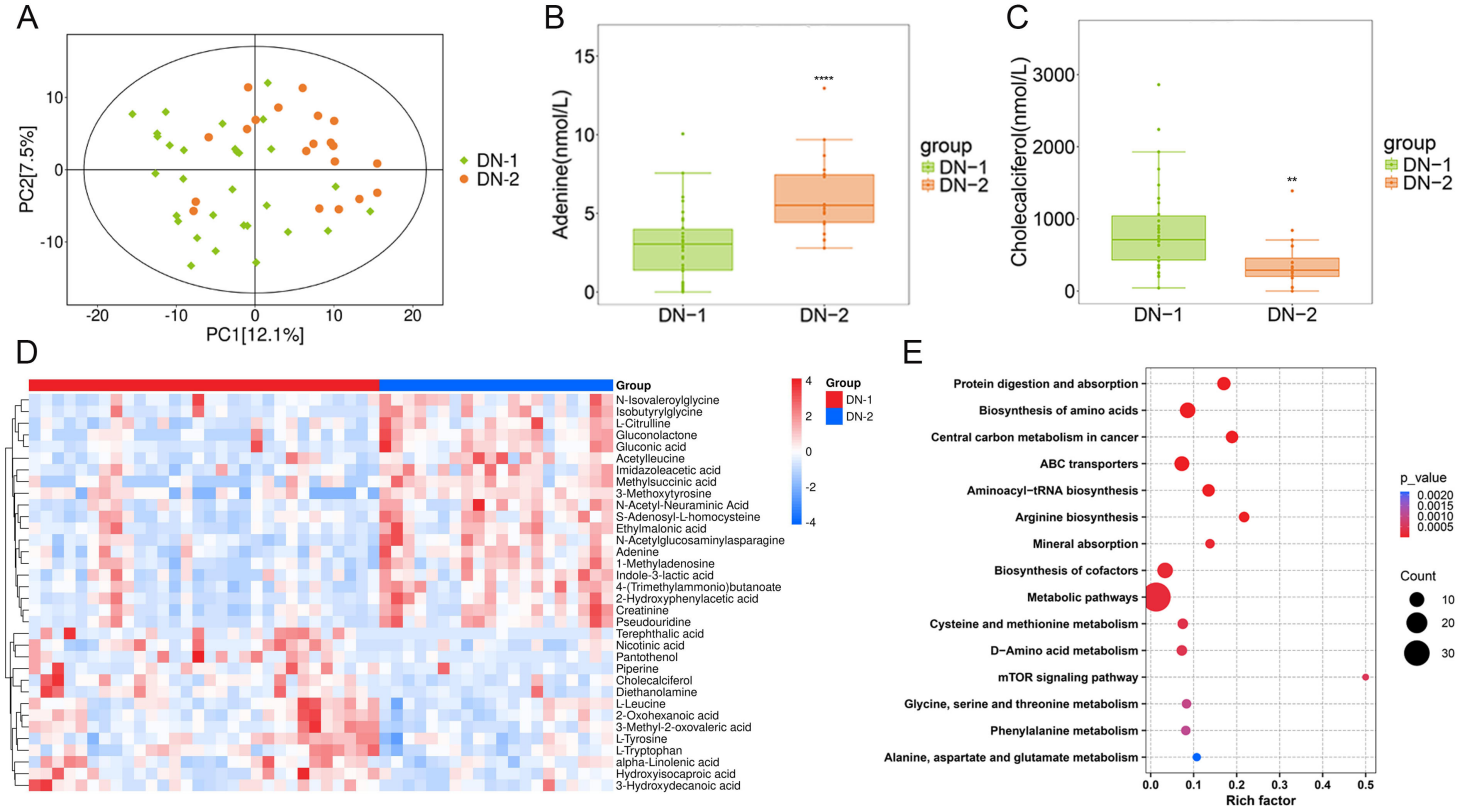
Metabolites composition and differences between groups with different degrees of renal injury. **(A)**. PCA analysis of DN-1 vs. DN-2; **(B, C)**. Expression levels of differential metabolites in different groups; **(D)**. Heat map of the top 20 differential metabolites; **(E)**. KEGG enrichment analysis of DN-1 vs. DN-2. *P < 0.05, **P < 0.01, ***P < 0.001, ****P < 0.0001.

To investigate whether metabolic profiles varied among groups with different degrees of renal damage, we analyzed the metabolomic data from the DN-1 and DN-2 groups. In the PCA plot, while the DN-2 group did not fully separate from the DN-1 group, distinct distribution trends were observed between them (**Figure 3A**). A total of 71 differentially expressed metabolites were identified in the DN-2 group compared with the DN-1 group, including 57 up-regulated metabolites and 14 down-regulated metabolites. Information regarding the top 20 metabolites that exhibited statistically significant differences in the studies is detailed in **Supplementary Table S3**. A heatmap illustrated the top 20 significantly differential metabolites identified when comparing the DN-1 group to the DN-2 group (**Figure 3D**). Among these, adenine and cholecalciferol, two metabolites associated with DN, were selected for further analysis, and their expression levels across the groups are presented in the **Figures 3B, C**. KEGG enrichment analysis revealed the top 15 differential pathways, including “Protein digestion and absorption”, “mTOR signaling pathway” and “Phenylalanine metabolism” among others (**Figure 3E**).

## 4 Discussion

The present study provides valuable insights into the demographic and biochemical characteristics of patients with T2DM and DN. The significant differences observed in age, smoking, drinking, and biochemical markers such as uACR, u-AlB, CREA, and eGFR between the control, DM, and DN groups highlight the progressive nature of kidney damage in DN. These findings underscore the importance of early detection and intervention in managing T2DM to prevent the onset and progression of DN.

### 4.1 Metabolic alterations in DM

Metabolomics has been extensively utilized as a powerful technique to identify potential biomarkers for disease diagnosis and to elucidate the underlying mechanisms of disease occurrence (17, 21–25). To identify the metabolic targets between patients with diabetes, diabetic nephropathy, and healthy controls, this study employed quantitative targeted metabolomics. The results demonstrated that the metabolic profiles of both diabetic and diabetic nephropathy patients were markedly distinct from those of healthy controls. Lactate, as the end product of glycolysis, plays a crucial role in maintaining acid-base balance and energy metabolism within the body. Our data revealed significantly elevated lactate levels in the diabetic groups compared to the control group (**Figure 1J**), with a trend toward higher lactate levels in the DN-1 group relative to the DM group. Extensive research has established that patients with diabetic nephropathy exhibit abnormal lactate metabolism, and there is a significant correlation between urinary lactate levels and renal tubular injury (26). In DN, alterations in renal energy metabolism, such as mitochondrial dysfunction and impaired fatty acid oxidation, significantly influence disease progression (27, 28). An abnormal increase in lactate levels, potentially resulting from renal metabolic disorders, may contribute to the progressive renal injury observed in DN (26). Other study has demonstrated that lactic acid can drive epithelial-mesenchymal transition of DN through H3K14la/KLF5 pathway, and aggravate renal tubular fibrosis in patients with DN (29). The findings of these studies are in high consistency with the results of the present study, underscoring the critical role of lactate metabolism in the progression of diabetic kidney injury. In the present study, L-ornithine levels were significantly elevated in the disease group relative to the control group (**Figure 1K**). As a key amino acid in the urea cycle, L-ornithine plays an essential role in ammonia detoxification by facilitating its conversion to urea, thereby reducing blood ammonia levels (30). In DN, the impaired filtration and excretion functions of the kidney may affect the efficiency of the urea cycle, consequently influencing the metabolism of L-ornithine (31). The progression of DN is closely linked to a chronic inflammatory response. As a precursor of nitric oxide (NO), arginine plays a crucial role in regulating vascular tone and modulating immune responses. In DN, aberrant arginine metabolism can result in diminished NO production, potentially impairing renal blood flow and exacerbating the inflammatory state (32). L-ornithine is a metabolite of arginine and may indirectly influence the production of inflammatory mediators by modulating arginine metabolism. Notably, our metabolomic enrichment analysis revealed significant alterations in arginine and proline metabolism as well as arginine biosynthesis in each disease group relative to the control group (**Figures 1G-I**). These findings indicate that L-ornithine could impact arginine metabolism, resulting in decreased NO production, which in turn affects renal blood supply, exacerbates the inflammatory response and interstitial fibrosis, and ultimately contributes to kidney injury. Additionally, these results suggest that lactate and L-ornithine may serve as important biomarkers and therapeutic targets for DM and DN.

### 4.2 Key metabolites specific to DN

To further investigate potential metabolic differences between DN and DM patients, the metabolomic profiles of the DN-1 and DN-2 groups were compared with those of the DM group. The results demonstrated that the metabolic signatures of DN patients were markedly distinct from those of DM patients, particularly in amino acid metabolism. Tryptophan, an essential aromatic amino acid, plays a multitude of critical physiological roles in the human body. Our study demonstrated that L-tryptophan levels were significantly lower in DN groups compared to DM group (**Figure 2G**). Numerous studies have highlighted the importance of L-tryptophan in the early detection of DN. In a metabonomic analysis of serum and urine samples from 286 diabetic patients, Solini et al. found that the combination of c-glycotryptophan, pseudouridine, and acetyl-L-threonine was associated with a lower glomerular filtration rate (GFR) and enhanced the predictive value of clinical parameters (33). By analyzing the serum metabolite levels of 52 diabetic patients with chronic kidney disease across various stages, Zhou et al. discovered that tryptophan levels were significantly correlated with a rapid decline in GFR. Specifically, tryptophan levels decreased as renal lesions progressed, suggesting its potential as a prognostic marker for DN (34). These findings are consistent with our results. Furthermore, our findings revealed that L-alanine levels were significantly reduced in the DN groups relative to the DM group (**Figure 2H**). Co-administration of L-alanine and L-glutamine was observed to enhance renal function in alloxan-induced diabetic rats (35). In a separate study, L-alanine supplementation was demonstrated to significantly improve blood glucose levels and biochemical parameters, restore tissue antioxidant levels, and enhance liver and kidney function in alloxan-induced diabetic rats (36). L-alanine promoted insulin secretion in INS-1E cells across a range of concentrations, with the effect becoming more pronounced at higher doses. These findings suggest that L-alanine has a dose-dependent positive influence on insulin secretion function (37). These findings suggest that L-alanine may be implicated in the development and progression of DN through multiple mechanisms, including alterations in amino acid metabolism and improvements in renal function and insulin secretion. In the present study, adenine levels were significantly elevated in the DN group compared to the DM group (**Figure 2I**). Our data indicate that adenine plays a crucial role in the progression of DN. For instance, adenine has been shown to induce kidney damage in mouse and rat models of chronic kidney disease (38, 39). Renal pathological changes induced by adenine administration encompass glomerular sclerosis, renal tubular atrophy, interstitial fibrosis, and inflammatory cell infiltration (40, 41). The expression patterns of these metabolites across groups are detailed in **Supplementary Table S3**. These findings indicate that L-tryptophan, L-alanine, and adenine could serve as potential biomarkers for the early diagnosis of DN.

### 4.3 Key metabolites specifically associated with the progression of DN

To further investigate the metabolic markers that could predict DN progression, we conducted an in-depth analysis of the metabolic profiles of the DN-1 and DN-2 groups, revealing significant alterations in several metabolites. Previous studies have confirmed that adenine effectively activates the mTOR pathway (42), and inhibiting mTOR can prevent renal lesions induced by adenine (43, 44). Sharma et al. utilized spatial metabolomics and single-cell transcriptomics of human kidney biopsies to demonstrate that adenine is specifically localized in the diseased areas of blood vessels, renal tubules, and glomeruli in diabetic patients. This finding suggests that adenine may serve as a potential endogenous pro-fibrotic factor. By stimulating the mTOR pathway, adenine could enhance the production of extracellular matrix by renal tubular cells, which is closely associated with the progression of DN. These results indicate that adenine may be a promising biomarker for DN (45), aligning with our study’s findings. Our study revealed that the mTOR pathway was significantly dysregulated in the DN-2 group compared to the DN-1 group (**Figure 3E**), potentially due to the markedly elevated adenine levels observed in the DN-2 group (**Figure 3B**). Interestingly, in the present study, cholecalciferol levels were significantly lower in the DN-2 group compared to the DN-1 group (**Figure 3C**), which may be associated with the progression of DN. Top differential metabolites are shown in **Figure 3D**. Previous research has indicated that cholecalciferol exerts a protective effect on renal function (46, 47). Agarwal et al. conducted a double-blind, randomized, placebo-controlled trial to evaluate the safety and efficacy of oral paricalcitol in patients with stage 3–4 secondary chronic kidney disease. The study randomized participants to receive either oral paricalcitol or placebo. Patients with nephropathy who received paricalcitol demonstrated a significant reduction in proteinuria excretion (48). These findings indicate that adenine and cholecalciferol hold promise as potential biomarkers for predicting the progression of DN and novel therapeutic agents for mitigating the progression of DN.

Overall, this study systematically identified several key metabolites with potential for the early diagnosis of DM and its complication, DN. Specifically, six metabolites were found to exhibit significant alterations in both DM and DN: lactic acid, L-ornithine, L-tryptophan, L-alanine, adenine, and cholecalciferol. Among these, lactic acid and L-ornithine may serve as potential biomarkers for the early detection of DM. Further analysis revealed that changes in L-tryptophan, L-alanine, adenine, and cholecalciferol were independent of the underlying pathological state of DM and were strongly associated with the onset and progression of DN. Notably, L-tryptophan and L-alanine could potentially act as biomarkers for the early diagnosis of DN, whereas adenine and cholecalciferol not only hold promise as indicators for predicting DN progression but may also represent novel therapeutic targets for mitigating disease advancement. Collectively, these metabolites can be considered specific markers reflecting kidney injury and provide critical insights for the early intervention of DN.

### 4.4 Limitations

However, our study has some limitations. While we utilized quantitative targeted metabolomics to analyze metabolites, resource and technical constraints prevented us from implementing stringent quality control measures to further validate our results. We acknowledge the importance of external validation to enhance the reliability and robustness of our findings. Therefore, future studies will aim to externally validate our results using independent cohorts, and we plan to conduct surveys with larger sample sizes to further confirm our findings. Additionally, the average age of patients with DN was significantly higher than that of the healthy control group. This primarily reflects the inherent nature of DN as a chronic progressive disease, where the duration of the disease naturally extends as the condition worsens. With increasing severity of DN, both the disease course and patient age tend to increase correspondingly. The “disease course-age” association is a well-documented phenomenon in chronic disease cohort studies, which poses significant challenges for achieving perfect age matching between DN patients and healthy controls in cross-sectional studies. Future research employing prospective designs or incorporating more precisely age-matched control groups could help further validate these findings.

## 5 Conclusion

This study utilized a comprehensive metabolomics approach to elucidate metabolic alterations in patients with T2DM and DN compared to healthy controls. The results demonstrated significant differences in the metabolic profiles of these groups, particularly in key metabolites such as lactate and L-ornithine, which were markedly elevated in T2DM and DN patients. These metabolites are involved in critical pathways including energy metabolism and the urea cycle, suggesting their potential utility as biomarkers for early detection and progression prediction of DM. Additionally, the study identified specific amino acids like L-tryptophan and L-alanine, adenine, and cholecalciferol, whose altered levels in DN patients may serve as indicators of disease severity and progression. The dysregulation of these metabolites in DN highlights the importance of metabolic interventions in managing the disease. These findings not only enhance our understanding of the pathophysiology of DN but also provide a foundation for developing novel diagnostic tools and therapeutic strategies aimed at mitigating the progression of DN complications.

## Data Availability

The original contributions presented in the study are included in the article/**Supplementary Material**. Further inquiries can be directed to the corresponding authors.
