# Supplementary material for: Identification of potential biomarkers for diabetic nephropathy via UPLC-MS/MS-based metabolomics
Source: Front Endocrinol (Lausanne). 2025 Sep 1;16:1581691. doi: 10.3389/fendo.2025.1581691 (PMC12433848; doi:10.3389/fendo.2025.1581691)
Supplement: Supplementary file 1 [file DataSheet1.docx]

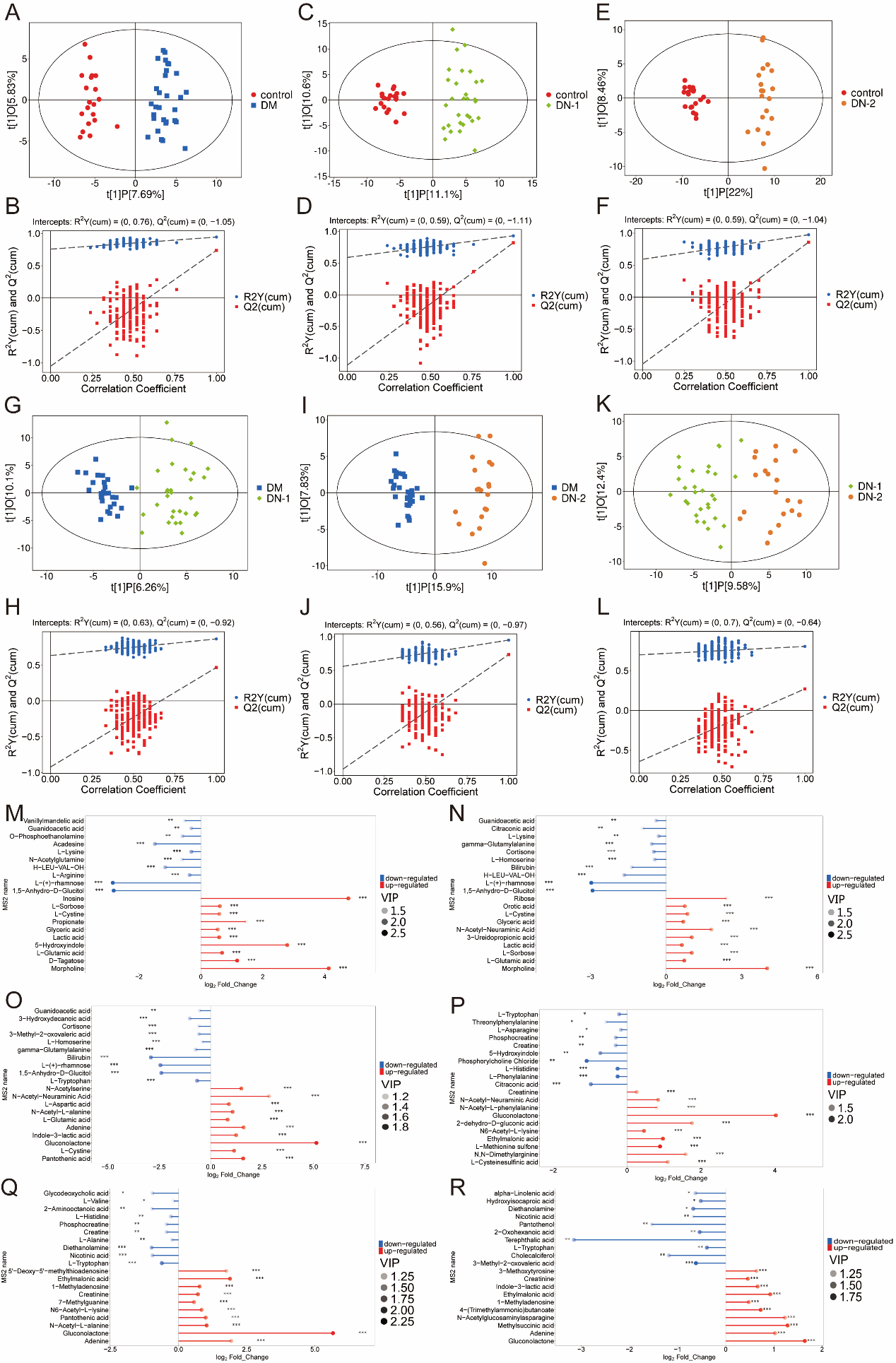


**FIGURE S1**

OPLS-DA analysis of pairwise comparisons among the control group, DM group, DN-1 group, and DN-2 group.

A-L. OPLS-DA analysis and permutation plots of Control vs. DM, Control vs. DN-1, Control vs. DN-2, DM vs. DN-1, DM vs. DN-2, and DN-1 vs DN-2 groups. M-R. VIP scores of metabolites from pairwise comparisons of different groups. Permutation plots (B, D, F, H, J and L) validate the corresponding OPLS-DA models (A, C, E, G, I and K). A negative Q^2^-intercept indicates that the original model is robust against overfitting.

OPLS-DA: orthogonal partial least squares discriminant analysis; VIP: variable importance in projection; DM: diabetes mellitus without nephropathy (urine albumin-to-creatinine ratio [uACR] < 30 mg/g); DN-1: diabetic nephropathy with microalbuminuria (uACR 30-300 mg/g); DN-2: diabetic nephropathy with macroalbuminuria (uACR > 300 mg/g). *P* < 0.05 was considered statistically significant.

**
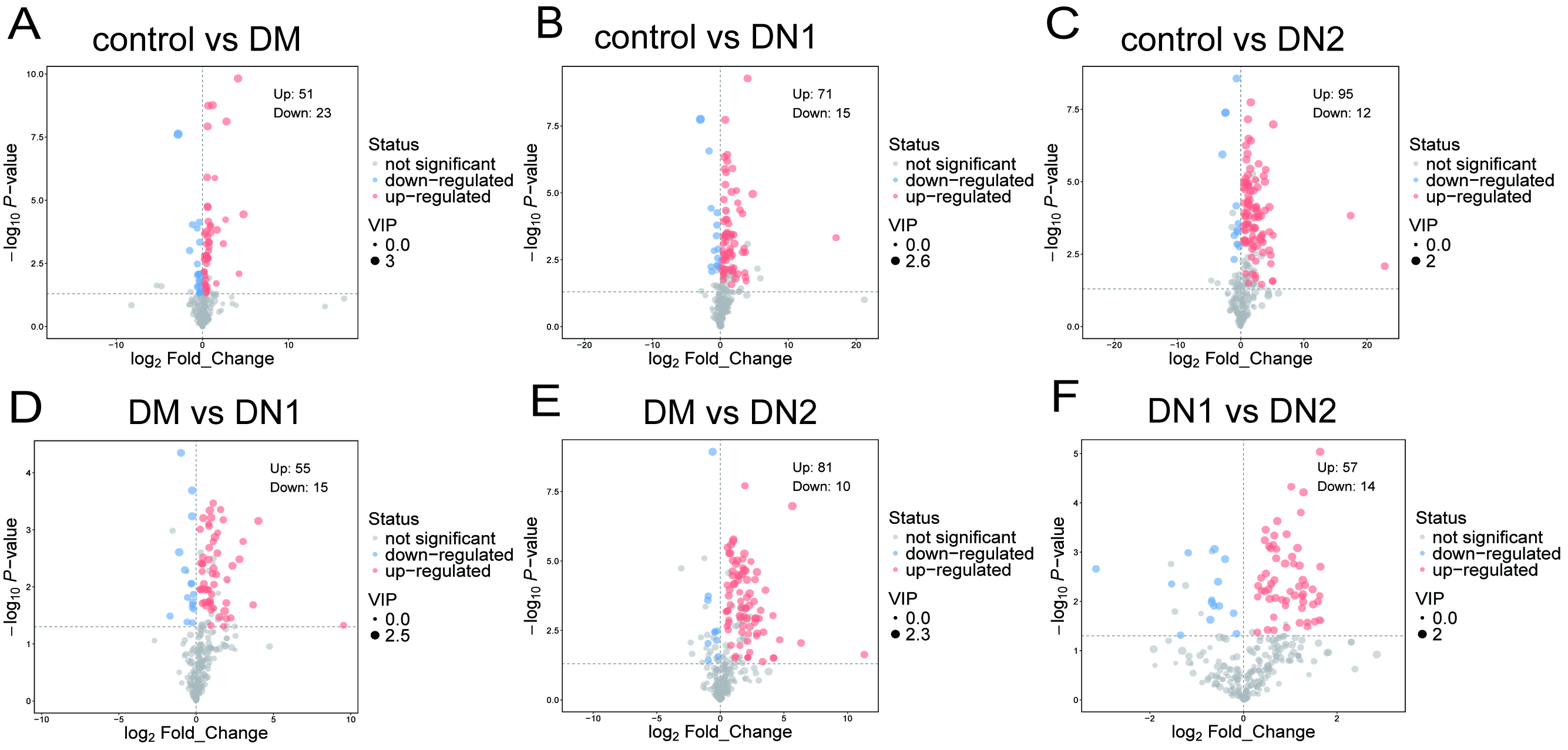
**

**FIGURE S2**

Volcano plots of differential metabolites between all comparison groups.

Volcano plot of differential metabolites between Control and DM groups. B. Volcano plot of differential metabolites between Control and DN-1 groups. C. Volcano plot of differential metabolites between Control and DN-2 groups. D. Volcano plot of differential metabolites between DM and DN-1 groups. E Volcano plot of differential metabolites between DM and DN-2 groups. F. Volcano plot of differential metabolites between DN-1 and DN-2 groups. DM: diabetes mellitus without nephropathy (urine albumin-to-creatinine ratio [uACR] < 30 mg/g); DN-1: diabetic nephropathy with microalbuminuria (uACR 30-300 mg/g); DN-2: diabetic nephropathy with macroalbuminuria (uACR > 300 mg/g). *P* < 0.05 was considered statistically significant.


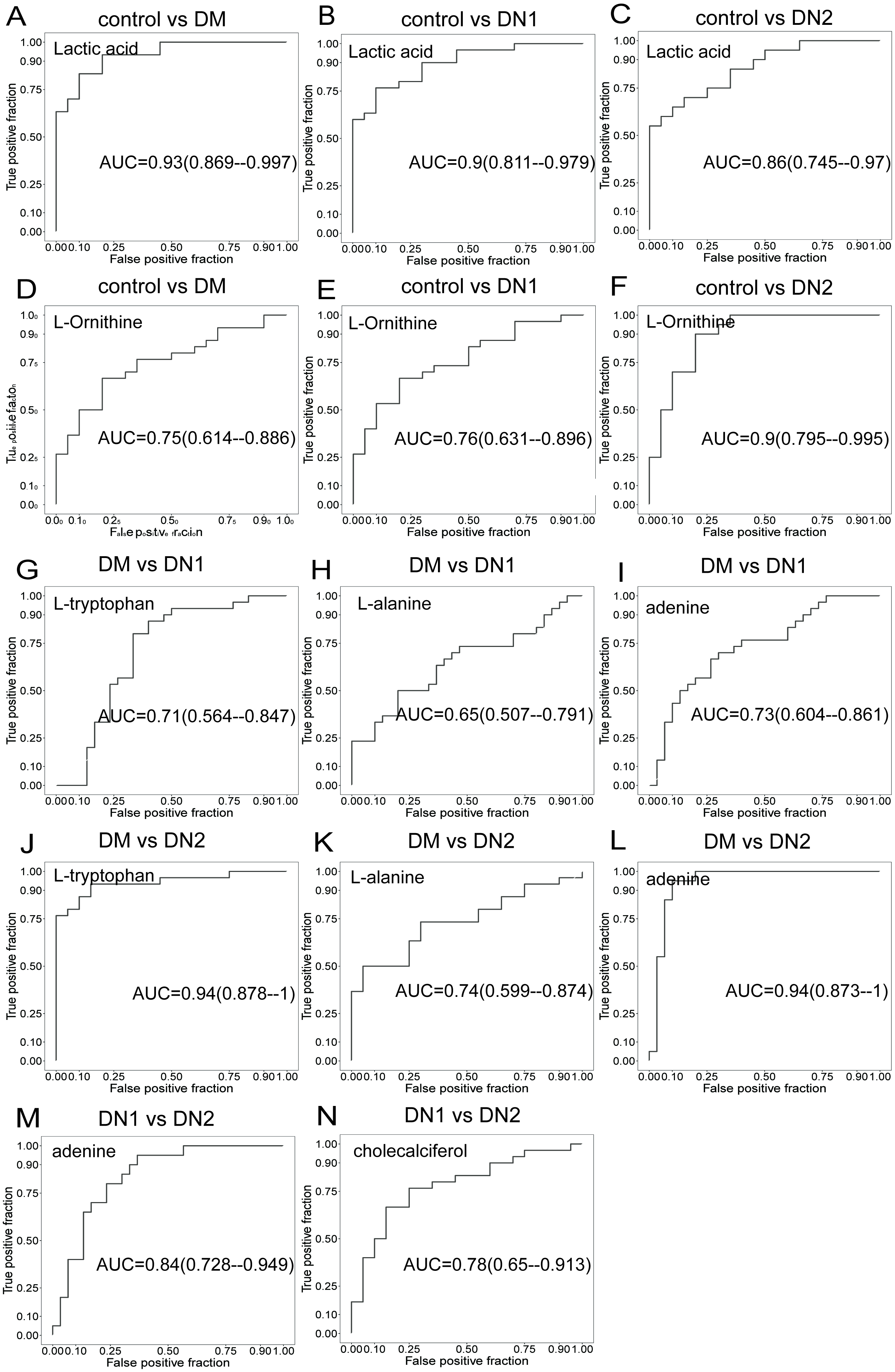


**FIGURE S3**

ROC curves for the 6 candidate biomarkers.

A-F. ROC curves for lactic acid and L-ornithine among Control vs. DM, Control vs. DN-1, Control vs. DN-2. G-L. ROC curves for L-tryptophan, L-alanine, adenine among DM vs. DN-1 and DM vs. DN-2. M-N. ROC curves for adenine, cholecalciferol between DN1 vs. DN-2. DM: diabetes mellitus without nephropathy (urine albumin-to-creatinine ratio [uACR] < 30 mg/g); DN-1: diabetic nephropathy with microalbuminuria (uACR 30-300 mg/g); DN-2: diabetic nephropathy with macroalbuminuria (uACR > 300 mg/g). *P* < 0.05 was considered statistically significant.
