# Supplementary material for: Identification of potential biomarkers for diabetic nephropathy via UPLC-MS/MS-based metabolomics
Source: Front Endocrinol (Lausanne). 2025 Sep 1;16:1581691. doi: 10.3389/fendo.2025.1581691 (PMC12433848; doi:10.3389/fendo.2025.1581691)
Supplement: Supplementary file 2 [file Table1.docx]

Table S1. The 12 internal standards used in metabolomics

| Number | Name | CAS |
| --- | --- | --- |
| 1 | L-(+)-Lactic Acid-13C3 Sodium Salt | 201595-71-3 |
| 2 | Anthranilic acid-[13C6] | 335081-06-6 |
| 3 | Succinic-2,2,3,3-d4 Acid | 14493-42-6 |
| 4 | N-Benzoyl-d5-glycine | 53518-98-2 |
| 5 | Octanoic-d15 Acid | 69974-55-6 |
| 6 | L-Glutamic-2.4.4-d3 Acid | 202468-31-3 |
| 7 | Nicotinamide-2,4,5,6-[d4] | 347841-88-7 |
| 8 | Acetylcholine-d9 Chloride (N,N,N-trimethyl-d9) | 344298-95-9 |
| 9 | L-Leucine-d3 (methyl-d3) | 87828-86-2 |
| 10 | N-(Carboxymethyl)-N,N,N-trimethyl-d9-ammonium Chloride | 285979-85-3 |
| 11 | Trimethyl-d9-amine N-Oxide | 1161070-49-0 |
| 12 | 4-Aminobutyric-2,2,3,3,4,4-d6 Acid | 70607-85-1 |

CAS: chemical abstracts service registry number.

Note: All internal standards were isotope-labeled analogs selected to represent major chemical classes of endogenous metabolites. Theses standards were used for normalization and quality control in LC-MS-based targeted metabolomics.

Table S2. 36 differential metabolites in DM compared to the normal group.

| Number | Class | Metabolite | p-value | VIP | log2FC |
| --- | --- | --- | --- | --- | --- |
| 1 | Others | Morpholine | <0.0001 | 2.7027 | 4.1298 |
| 2 | Amino acids, peptides, and analogues | L-Glutamic acid | <0.0001 | 2.6360 | 0.6841 |
| 3 | Carbohydrates and carbohydrate conjugates | D-Tagatose | <0.0001 | 2.8684 | 1.1724 |
| 4 | Indoles and derivatives | 5-Hydroxyindole | <0.0001 | 2.5485 | 2.7890 |
| 5 | Organic acids and derivatives | Lactic acid | <0.0001 | 2.4962 | 0.5907 |
| 6 | Carbohydrates and carbohydrate conjugates | L-(+)-rhamnose | <0.0001 | 2.9720 | -2.8455 |
| 7 | Carbohydrates and carbohydrate conjugates | 1,5-Anhydro-D-Glucitol | <0.0001 | 2.9664 | -2.8157 |
| 8 | Carbohydrates and carbohydrate conjugates | Glyceric acid | <0.0001 | 2.0872 | 0.5415 |
| 9 | Carbohydrates and carbohydrate conjugates | L-Sorbose | <0.0001 | 2.2770 | 0.6122 |
| 10 | Amino acids, peptides, and analogues | L-Cystine | <0.0001 | 1.9977 | 0.5950 |
| 11 | Nucleosides, nucleotides, and analogues | Inosine | <0.0001 | 2.6380 | 4.7632 |
| 12 | Carbohydrates and carbohydrate conjugates | Ribose | 0.0001 | 1.1144 | 2.6860 |
| 13 | Pyrimidines and pyrimidine derivatives | Orotic acid | 0.0001 | 1.9088 | 0.6813 |
| 14 | Carbohydrates and carbohydrate conjugates | N-Acetyl-Neuraminic Acid | 0.0001 | 1.5840 | 0.9568 |
| 15 | Carbohydrates and carbohydrate conjugates | D-Glucosamine | 0.0001 | 1.6780 | 0.8843 |
| 16 | Nucleosides, nucleotides, and analogues | Guanosine | 0.0001 | 2.0969 | 1.6929 |
| 17 | Amino acids, peptides, and analogues | L-Aspartic acid | 0.0002 | 1.9312 | 0.4380 |
| 18 | Amino acids, peptides, and analogues | Homocitrulline | 0.0002 | 1.8570 | 0.9520 |
| 19 | Nucleosides, nucleotides, and analogues | Uridine | 0.0002 | 1.9755 | 0.2774 |
| 20 | Organic acids and derivatives | Pantothenic acid | 0.0003 | 1.7877 | 0.5863 |
| 21 | Amino acids, peptides, and analogues | Cysteine-S-sulfate | 0.0004 | 1.9971 | 0.7356 |
| 22 | Biogenic Amines | N-Acetylputrescine | 0.0005 | 1.5323 | 2.4264 |
| 23 | Amino acids, peptides, and analogues | Guanidinosuccinic acid | 0.0018 | 1.8491 | 0.5995 |
| 24 | Organic acids and derivatives | 3-Ureidopropionic acid | 0.0019 | 1.7054 | 0.6050 |
| 25 | Amino acids, peptides, and analogues | L-Ornithine | 0.0023 | 1.4394 | 0.3651 |
| 26 | Amino acids, peptides, and analogues | gamma-L-Glutamyl-L-valine | 0.0033 | 1.4505 | 0.4887 |
| 27 | Organic acids and derivatives | Urea | 0.0068 | 1.5852 | 0.2063 |
| 28 | Amino acids, peptides, and analogues | Guanidoacetic acid | 0.0082 | 1.4373 | -0.3127 |
| 29 | Carbohydrates and carbohydrate conjugates | Mannose 6-phosphate | 0.0081 | 1.2627 | 4.2482 |
| 30 | Amino acids, peptides, and analogues | gamma-Glutamylalanine | 0.0154 | 1.4831 | -0.3979 |
| 31 | Others | Inositol | 0.0214 | 1.3762 | 0.3183 |
| 32 | Amino acids, peptides, and analogues | L-Homoserine | 0.0239 | 1.3357 | -0.2124 |
| 33 | Amino acids, peptides, and analogues | 5-Hydroxylysine | 0.0235 | 1.2142 | 0.3727 |
| 34 | Benzenoids | 2-Hydroxyphenylacetic acid | 0.0261 | 1.1778 | 0.5593 |
| 35 | Steroids and steroid derivatives | Cortisone | 0.0432 | 1.0288 | -0.2455 |
| 36 | Organic acids and derivatives | beta-Hydroxyisovaleric acid | 0.0478 | 1.2845 | 0.4291 |

VIP: variable importance in projection; log2FC: log2 fold change; DM: diabetes mellitus.

Note: Metabolites were identified as significantly different between DM and normal groups using multivariate (PLS-DA) and univariate statistical analysis. VIP values were derived from PLS-DA modeling. Log2FC indicates the binary logarithm of fold change (DM vs. normal group).

Statistical significance: p-values were calculated using the Wilcoxon rank-sum test; *P* < 0.05 was considered statistically significant.

Table S3. 50 deferentially expressed metabolites in DN-1 and DN-2 vs. DM group.

| Number | Class | Metabolite | p-value | VIP | log2FC |
| --- | --- | --- | --- | --- | --- |
| 1 | Amino acids, peptides, and analogues | L-Tryptophan | <0.0001 | 1.9659 | -0.6095 |
| 2 | Purines and purine derivatives | Adenine | <0.0001 | 1.3775 | 1.9341 |
| 3 | Carbohydrates and carbohydrate conjugates | Gluconolactone | <0.0001 | 2.2786 | 5.6635 |
| 4 | Amino acids, peptides, and analogues | N-Acetyl-L-alanine | <0.0001 | 2.0529 | 1.0326 |
| 5 | Amino acids, peptides, and analogues | N6-Acetyl-L-lysine | <0.0001 | 1.7825 | 0.8444 |
| 6 | Purines and purine derivatives | 7-Methylguanine | <0.0001 | 1.7121 | 0.5694 |
| 7 | Amino acids, peptides, and analogues | Creatinine | <0.0001 | 1.9386 | 0.7131 |
| 8 | Nucleosides, nucleotides, and analogues | 1-Methyladenosine | <0.0001 | 1.8575 | 0.7748 |
| 9 | Organic acids and derivatives | Ethylmalonic acid | <0.0001 | 2.0334 | 1.8916 |
| 10 | Nucleosides, nucleotides, and analogues | 5'-Deoxy-5'-methylthioadenosine | <0.0001 | 1.3246 | 1.7563 |
| 11 | Amino acids, peptides, and analogues | N-Acetylserine | <0.0001 | 1.9842 | 1.3207 |
| 12 | Amino acids, peptides, and analogues | N,N-Dimethylarginine | <0.0001 | 1.1353 | 2.4082 |
| 13 | Nucleosides, nucleotides, and analogues | N4-Acetylcytidine | <0.0001 | 1.7285 | 1.2332 |
| 14 | Amino acids, peptides, and analogues | N-Acetylglutamine | <0.0001 | 1.1462 | 1.7843 |
| 15 | Carbohydrates and carbohydrate conjugates | N-Acetyl-Neuraminic Acid | <0.0001 | 1.9154 | 1.8788 |
| 16 | Nucleosides, nucleotides, and analogues | Pseudouridine | <0.0001 | 2.1103 | 1.9661 |
| 17 | Nucleosides, nucleotides, and analogues | S-Adenosyl-L-homocysteine | <0.0001 | 1.8441 | 0.9711 |
| 18 | Amino acids, peptides, and analogues | Homocitrulline | <0.0001 | 2.0017 | 2.8782 |
| 19 | Amino acids, peptides, and analogues | L-Pyroglutamic acid | <0.0001 | 1.8441 | 1.7092 |
| 20 | Carbohydrates and carbohydrate conjugates | Gluconic acid | <0.0001 | 2.0791 | 1.4571 |
| 21 | Amino acids, peptides, and analogues | L-Cysteinesulfinic acid | <0.0001 | 1.0634 | 1.4230 |
| 22 | Amino acids, peptides, and analogues | N-Acetyl-L-glutamic acid | <0.0001 | 1.4701 | 2.7305 |
| 23 | Benzenoids | 2-Hydroxyphenylacetic acid | <0.0001 | 1.9372 | 2.0995 |
| 24 | Nucleosides, nucleotides, and analogues | N,N-Dimethylguanosine | 0.0001 | 1.9206 | 1.0989 |
| 25 | Organic acids and derivatives | Urea | 0.0001 | 1.6896 | 0.7353 |
| 26 | Carbohydrates and carbohydrate conjugates | D-Glucuronate | 0.0001 | 2.1715 | 2.3051 |
| 27 | Pyridines and derivatives | Trigonelline | 0.0002 | 1.7791 | 2.0933 |
| 28 | Amino acids, peptides, and analogues | D-Alanyl-D-alanine | 0.0003 | 1.4647 | 2.6535 |
| 29 | Pyridines and derivatives | Quinolinic acid | 0.0004 | 1.9432 | 2.2737 |
| 30 | Pyridines and derivatives | 4-Pyridoxic acid | 0.0005 | 1.8975 | 1.9594 |
| 31 | Organic acids and derivatives | 2-Isopropylmalic acid | 0.0006 | 1.3823 | 3.1711 |
| 32 | Amino acids, peptides, and analogues | N,N-Dimethylglycine | 0.0006 | 1.4570 | 0.5728 |
| 33 | Amino acids, peptides, and analogues | N-Formylglycine | 0.0006 | 1.4570 | 0.5728 |
| 34 | Benzenoids | P-Cresol | 0.0009 | 1.2397 | 4.1371 |
| 35 | Amino acids, peptides, and analogues | L-Methionine sulfone | 0.0009 | 1.8775 | 1.5212 |
| 36 | Amino acids, peptides, and analogues | 4-Acetamidobutyric acid | 0.0011 | 2.0107 | 2.7884 |
| 37 | Others | Kynurenic acid | 0.0012 | 1.8620 | 2.0873 |
| 38 | Indoles and derivatives | 5-Hydroxyindole-3-acetic acid | 0.0012 | 1.1206 | 3.0709 |
| 39 | Organic acids and derivatives | 3-Hydroxy-3-methylglutaric acid | 0.0022 | 1.8137 | 1.7728 |
| 40 | Carbohydrates and carbohydrate conjugates | Erythritol | 0.0023 | 1.8826 | 2.3244 |
| 41 | Amino acids, peptides, and analogues | L-Alanine | 0.0033 | 1.1353 | -0.2357 |
| 42 | Amino acids, peptides, and analogues | Creatine | 0.0036 | 1.1660 | -0.4354 |
| 43 | Amino acids, peptides, and analogues | Phosphocreatine | 0.0036 | 1.1650 | -0.4341 |
| 44 | Carbohydrates and carbohydrate conjugates | 2-dehydro-D-gluconic acid | 0.0036 | 1.6487 | 3.3838 |
| 45 | Amino acids, peptides, and analogues | Guanidinosuccinic acid | 0.0040 | 1.8578 | 2.9032 |
| 46 | Carbohydrates and carbohydrate conjugates | Mannitol | 0.0069 | 1.2797 | 4.6658 |
| 47 | Amino acids, peptides, and analogues | L-Histidine | 0.0071 | 1.2695 | -0.2776 |
| 48 | Amino acids, peptides, and analogues | 2-Aminooctanoic acid | 0.0092 | 1.0968 | -0.9660 |
| 49 | Amino acids, peptides, and analogues | O-Benzyl-L-serine | 0.0235 | 1.5013 | 11.3225 |
| 50 | Amino acids, peptides, and analogues | Methionine sulfoxide | 0.0308 | 1.2209 | 1.1503 |

VIP: variable importance in projection; log2FC: log2 fold change; DM: diabetes mellitus without nephropathy (urine albumin-to-creatinine ratio [uACR] < 30 mg/g); DN-1: diabetic nephropathy with microalbuminuria (uACR 30–300 mg/g); DN-2: diabetic nephropathy with macroalbuminuria (uACR > 300 mg/g).

Note: Metabolites were identified as differentially expressed in DN-1 and DN-2 compared to DM using multivariate (PLS-DA) and univariate (Wilcoxon rank-sum) analyses. VIP scores were obtained from PLS-DA models, and log2FC values indicate the binary logarithm of fold change relative to the DM group.

Statistical significance: *P* < 0.05 was considered statistically significant.

Table S4. The top 20 metabolite of DN-1 vs DN-2.

| Number | Class | Metabolite | p-value | VIP | log2FC |
| --- | --- | --- | --- | --- | --- |
| 1 | Carbohydrates and carbohydrate conjugates | Gluconolactone | <0.0001 | 1.8901 | 1.6397 |
| 2 | Purines and purine derivatives | Adenine | <0.0001 | 1.4752 | 1.0210 |
| 3 | Organic acids and derivatives | Methylsuccinic acid | 0.0001 | 1.8249 | 1.2830 |
| 4 | Carbohydrates and carbohydrate conjugates | N-Acetylglucosaminylasparagine | 0.0002 | 1.4019 | 1.2263 |
| 5 | Biogenic Amines | 4-(Trimethylammonio)butanoate | 0.0002 | 1.8264 | 0.7244 |
| 6 | Nucleosides, nucleotides, and analogues | 1-Methyladenosine | 0.0004 | 1.5607 | 0.4749 |
| 7 | Organic acids and derivatives | Ethylmalonic acid | 0.0004 | 1.7151 | 0.9229 |
| 8 | Indoles and derivatives | Indole-3-lactic acid | 0.0005 | 1.6121 | 0.6602 |
| 9 | Amino acids, peptides, and analogues | Creatinine | 0.0006 | 1.6751 | 0.4565 |
| 10 | Amino acids, peptides, and analogues | 3-Methoxytyrosine | 0.0007 | 1.3504 | 0.6361 |
| 11 | Others | Imidazoleacetic acid | 0.0008 | 1.6078 | 0.5499 |
| 12 | Nucleosides, nucleotides, and analogues | Pseudouridine | 0.0008 | 2.0388 | 1.1563 |
| 13 | Nucleosides, nucleotides, and analogues | S-Adenosyl-L-homocysteine | 0.0008 | 1.4689 | 0.5846 |
| 14 | Amino acids, peptides, and analogues | Acetylleucine | 0.0009 | 1.2808 | 0.7079 |
| 15 | Organic acids and derivatives | 3-Methyl-2-oxovaleric acid | 0.0009 | 1.9713 | -0.6200 |
| 16 | Steroids and steroid derivatives | Cholecalciferol | 0.0010 | 1.2795 | -1.1802 |
| 17 | Carbohydrates and carbohydrate conjugates | N-Acetyl-Neuraminic Acid | 0.0012 | 1.8288 | 1.0398 |
| 18 | Amino acids, peptides, and analogues | L-Citrulline | 0.0013 | 1.6323 | 0.6990 |
| 19 | Amino acids, peptides, and analogues | L-Tryptophan | 0.0014 | 1.6927 | -0.3928 |
| 20 | Carbohydrates and carbohydrate conjugates | Gluconic acid | 0.0017 | 1.8990 | 0.8900 |

VIP: variable importance in projection; log2FC: log2 fold change; DN-1: diabetic nephropathy with microalbuminuria (uACR 30–300 mg/g); DN-2: diabetic nephropathy with macroalbuminuria (uACR > 300 mg/g).

Note: Metabolites were identified as differentially expressed in DN-1 and DN-2 compared to DM using multivariate (PLS-DA) and univariate (Wilcoxon rank-sum) analyses. VIP scores were obtained from PLS-DA models, and log2FC values indicate the binary logarithm of fold change relative to the DM group.

Statistical significance: *P* < 0.05 was considered statistically significant.
